# Clinical characteristics of neonatal and infant osteomyelitis and septic arthritis: a multicenter retrospective study

**DOI:** 10.1016/j.jped.2024.03.003

**Published:** 2024-04-17

**Authors:** Keming Sun, Chunxu Zhang, Ziwen Mao, Chen Wang, Hua Zhu, Huiqing Sun, Kang Wang, Weyland Cheng

**Affiliations:** a Children's Hospital Affiliated to Zhengzhou University, Henan Children's Hospital, Zhengzhou Children's Hospital, Department of Orthopaedic Surgery, Zhengzhou, Henan, China; b Children's Hospital Affiliated to Zhengzhou University, Henan Children's Hospital, Zhengzhou Children's Hospital, Henan Provincial Key Laboratory of Children's Genetics and Metabolic Diseases, Zhengzhou, Henan, China; c Hebei Children's Hospital, Department of Orthopaedic Surgery, Shijiazhuang, Hebei, China; d Children's Hospital Affiliated to Zhengzhou University, Henan Children's Hospital, Zhengzhou Children's Hospital, Department of Neonatology, Zhengzhou, Henan, China

**Keywords:** Osteomyelitis, Neonates, Infants, Septic arthritis, Clinical characteristics

## Abstract

**Objective:**

Signs and symptoms of osteomyelitis or septic arthritis in neonates and infants are often nonspecific and early-stage bone infections in infants may often go unnoticed. The objective of this study was to analyze the clinical characteristics of newborns and infants with osteomyelitis and septic arthritis to improve understanding of the disorder and to assist clinicians with diagnosis.

**Methods:**

A retrospective multicenter study was conducted on neonates (0–28 days old, *n* = 94) and infants (1–12 months old, *n* = 415) with osteoarticular infections. Data consisting of clinical characteristics, complications, laboratory outcomes, and the pathogenic microorganisms causing osteomyelitis were tabulated. The statistics were further broken down into two regions and the significant differences between neonates and infants were evaluated and compared to the literature.

**Results:**

Compared to infants, neonates had significantly lower incidences of fever (*p* < 0.0001), higher incidences of localized swelling (*p* = 0.0021), higher rate of infection at the humerus (*p* = 0.0016), higher percentage of *Escherichia coli* (*p* < 0.0001) and *Klebsiella pneumoniae* (*p* = 0.0039) infections, lower percentage of *Staphylococcus aureus* infections (*p* < 0.0001) and were more likely to develop septic arthritis (*p* < 0.0001).

**Conclusion:**

Distinct differences were found between neonatal and infants with osteoarticular infections. Future studies should focus on improving diagnosis and subsequent treatment regimens for younger age groups.

## Introduction

Osteomyelitis or septic arthritis are bacterial, viral, or fungal infections of the bone or joint, respectively, that cause inflammation, which leads to debilitating consequences for both adults and children. If left untreated in children, osteoarticular infections can result in osteonecrosis, which potentially leads to fractures, limb-length discrepancy, sepsis or septic shock, avascular necrosis of the femoral head, fractures, deep vein thrombosis or in rare cases, death.^1^^,^^2^ Both osteomyelitis and septic arthritis are typically treated through antibiotics and often in addition to surgical debridement or drainage in complicated cases.^3^ In rare severe cases, amputation may be necessary. Due to the gravity of the infection, knowledge of epidemiological and clinical characteristics is essential to establish early diagnoses and to develop suitable treatment regimens, which are paramount to minimizing complications and optimizing outcomes.^4^ Identification of the causative pathogen is also required to confirm the diagnosis and optimize antibiotic therapy.^1^ It is thus essential for healthcare workers who care for neonates and infants in intensive care units to have an understanding of osteoarticular manifestations and be equipped to recognize early symptoms, to which they can initiate treatments to prevent long-term sequelae.^5^

The incidence rate of acute osteomyelitis and septic arthritis in neonatal intensive care units (NICUs) is approximately 1 to 3 cases and 0.12 cases per 1000 neonates, respectively.^6^^,^^7^ Neonates have been reported to have an increased risk of sepsis, which is a risk factor for the development of osteomyelitis and septic arthritis.^5^ The clinical presentation of osteoarticular infections in neonates and infants also differs compared to that of older children. Due to immature humoral and cellular defense mechanisms, neonates and infants do not have the typical inflammatory responses that are key indicators for an early diagnosis.^8^ As the signs and symptoms in infants can also be nonspecific, early-stage bone infections in infants may often go unnoticed and a late diagnosis by only 4 days is a risk factor for long-term sequelae.^9^

In neonates and infants, the development of osteoarticular infections is relatively rare and reports in the literature on neonatal infections can range from single case reports to case series of up to 77 cases.^10^, ^11^, ^12^, ^13^, ^14^, ^15^ In this study, the authors examine the clinical presentations of osteoarticular infections in neonates and infants, using data from multiple hospital centers. The objective of this study was to determine whether there were differences in the symptoms and prognosis of osteoarticular infections in neonates as compared to infants up to one year old, in order to facilitate clinicians in the identification and response to the disease. The importance of the clinical differences between neonates and infants is then discussed and compared to reports in the literature.

## Method

### *Patient population*

Data from 509 patients consisting of 94 neonates (0–28 days old) and 415 infants 1–12 months who were diagnosed with osteomyelitis or septic arthritis were collected between January 1, 2015 and December 31, 2022 from five hospitals: Hebei Children's Hospital (*n* = 346), Children's Hospital Affiliated to Zhengzhou University (*n* = 101), Xi'an Children's Hospital (*n* = 29), The Third Affiliated Hospital of Zhengzhou University (*n* = 23) and Beijing Children's Hospital (*n* = 10). Eligible patients for enrollment included term infants and preterm infants diagnosed with acute osteomyelitis or septic arthritis. Exclusion criteria included infants with genetic metabolic disease or congenital abnormalities, chronic osteomyelitis, recurrent cases, and extreme preterm cases (< 28 weeks).

### *Study ethics*

The study was approved by the Ethics Review Committee of all five hospitals. Informed consent was waived as this was a retrospective study and all identifiable personal data was omitted. The study was conducted in accordance with the Declaration of Helsinki for human subjects.

### *Data collection and study outcomes*

Patient and diagnostic characteristics consisting of age, sex, region of infection, side of infection, fever, localized erythema, localized swelling, apparent pain, warmth at the site and lack of spontaneous movement were collected. Study-specific variables were also collected retrospectively, including bacterial culture, surgical management, complications, and laboratory test results. Complications included sepsis, pneumonia, localized cellulitis, and septic arthritis. Laboratory markers consisted of peak white blood cell (WBC) count, platelet (PLT) count, c-reactive protein (CRP), and erythrocyte sedimentation rate (ESR). The results were further broken down into two regions (Henan and Hubei province) based on the locations of the hospitals.

### *Statistical analysis*

Log-normal distribution was used to determine the normality of the data, which was confirmed through the D'Agostino & Pearson, Shapiro-Wilk, and Kolmogorov-Smirnov tests. Infant characteristics and outcomes were compared using Fisher's exact test for categorical variables and Mann-Whitney U for non-parametric continuous variables (log-normal distribution of all numerical variables were non-normal in at least one group). Crude relative risks (RR) with 95% confidence intervals (95% CIs) were estimated for patient characteristics. The level of statistical significance was set at *p* < 0.05. Graphpad Prism 9.0 was used for statistical analysis and production of graphs.

## Results

### *Patient and diagnostic characteristics*

Table 1 displays the patient characteristics and initial symptoms of the infants and neonates. Neonates presented with significantly lower incidences of fever (*p* < 0.0001), and significantly higher incidences of local swelling (*p* = 0.0021), warmth at the site of infection (*p* = 0.0018), and lack of spontaneous movement (*p* = 0.0002). In both infants and neonates, there was a higher ratio of males compared to females. When the statistics were examined by province, warmth at the site of infection was significantly higher in neonates in Henan (*n* = 126), but the same trend did not hold true for Hebei (346). Contrarily, the lack of spontaneous movement was significantly higher in neonates within Hebei, but not Henan.

**Table 1:** Clinical characteristics of osteomyelitis in neonates and infants.

	**Infant (*n*=415)**	**Neonate (*n*=94)**	**Relative Risk**	***P*-value**^a^
**Age (mo), mean ± SD**	5.7 ± 3.8	0.64 ± 0.25		<0.0001
**Male/Female, n (%)**	243 (58.6) / 172 (41.4)	59 (62.8) / 39 (37.2)	1.155 (0.7954 – 1.690)	0.4529
**Fever, n (%)**	300 (72.3)	43 (45.7)	0.4080 (0.2852 – 0.5853)	<0.0001
**Localized erythema, n (%)**	142 (34.2)	33 (35.1)	1.033 (0.7028 – 1.502)	0.8698
**Localized swelling, n (%)**	368 (88.7)	93 (98.9)	9.683 (1.834 – 55.04)	0.0021
**Warmth at site, n (%)**	287 (69.2)	80 (85.1)	2.211 (1.321 – 3.780)	0.0018
**Lack of spontaneous movement, n (%)**	331 (79.8)	90 (95.7)	4.703 (1.889 – 12.2)	0.0002
**Side of Infection: Left/Right, n (%)**	214 (51.6) / 201 (48.4)	52 (55.3) / 42 (44.7)	1.028 (0.9453 – 1.842)	0.5107
**Henan Province (infant, *n*=77; neonate, *n*=47)**				
**Age (mo), mean ± SD**	7.84 ± 3.63	0.62 ± 0.28		<0.0001
**Male/Female, n (%)**	47 (61.0) / 30 (39.0)	30 (63.8) / 17 (36.2)	1.077 (0.6838 – 1.751)	0.7560
**Fever, n (%)**	63 (81.8)	24 (51.1)	0.4438 (0.2914 – 0.6826)	0.0003
**Localized erythema, n (%)**	16 (20.8)	13 (27.7)	1.253 (0.7450 – 1.963)	0.3799
**Localized swelling, n (%)**	71 (92.2)	47 (100)	Infinity (1.001 – infinity)	0.0819^b^
**Warmth at site, n (%)**	53 (68.8)	47 (100)	Infinity (3.386 – infinity)	<0.0001^b^
**Lack of spontaneous movement, n (%)**	76 (98.7)	47 (100)	Infinity (0.4627 – infinity)	0.4328
**Side of Infection: Left/Right, n (%)**	49 (63.6) / 28 (36.4)	29 (61.7) / 18 (38.3)	0.9689 (0.7108 – 1.277)	0.8287
**Hebei Province (infant, *n*=302; neonate, *n*=44)**				
**Age (mo), mean ± SD**	5.12 ± 3.63	0.65 ± 0.22		
**Male/Female, n (%)**	171 (56.6) / 131 (43.38)	28 (63.6) / 16 (36.4)	1.293 (0.7356 – 2.293)	0.3792
**Fever, n (%)**	207 (68.54)	19 (43.2)	0.4035 (0.2337 – 0.6986)	0.0010
**Localized erythema, n (%)**	126 (41.72)	19 (43.2)	1.054 (0.6058 – 1.821)	0.1834
**Localized swelling, n (%)**	263 (87.1)	43 (97.7)	5.621 (1.067 – 32.19)	0.0392
**Warmth at site, n (%)**	302 (66.6)	30 (68.2)	1.067 (0.5992 – 1.930)	0.8307
**Lack of spontaneous movement, n (%)**	220 (72.9)	40 (90.9)	3.308 (1.297 – 8.749)	0.0096
**Side of Infection: Left/Right, n (%)**	158 (52.3) / 144 (47.7)	22 (50) / 22 (50)	0.9883 (0.9078 – 1.073)	0.7737

a All statistics were conducted using the Mann-Whitney U comparison or Chi-square test unless otherwise indicated.

b Fisher's exact test.

**Figure 1:**
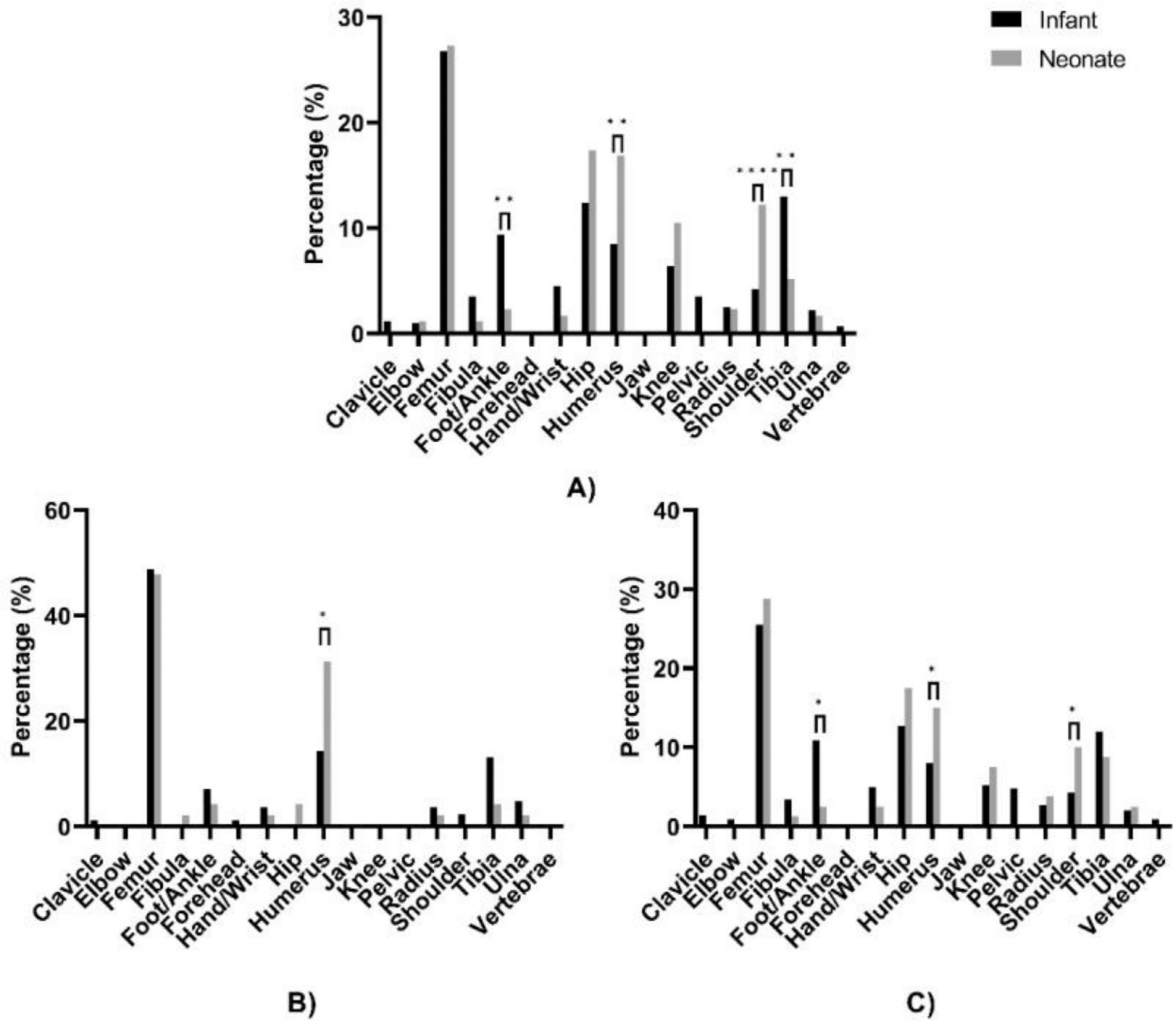
Regions of bacterial infection in neonates and infants with acute osteoarticular infections; (A) All patients, (B) patients from Henan province, (C) patients from Hebei province.

In terms of region of infection (Figure 1A), neonates had significantly less infections in the foot/ankle (4/172, 2.3%) and tibia (9/172, 5.2%) compared to infants (56/598, 9.4% and 78/598, 13.0%) with *p*-values of 0.0024 and 0.0043, respectively. Furthermore, neonates had a significantly higher distribution of infections in the humerus (29/172, 16.9%) and shoulder (21/172, 12.2%) compared to infants (51/598, 8.5% and 25/598, 4.2%) with *p*-values of 0.0016 and < 0.0001, respectively. Neonates had the highest overall percentage of infections in the femur, hip and humerus (27.3%, 17.4%, and 16.9% overall, respectively) whereas infants had the highest number of infections in the femur, tibia and hip (26.8%, 13.0% and 12.4% overall, respectively).

When the regions of infection were further examined by province (Figure 1B, C), neonates only had higher incidences of infection in the humerus in Henan whereas there were significant differences in the frequency of infections at the foot/ankle, humerus, and shoulder between neonates and infants within Hebei province.

### *Blood indicators and complications*

Neonates displayed significantly higher WBC (14.91 ± 5.76 10^9^/L), PLT (475.50 ± 191.42 10^9^/L) and lower CRP (32.34 ± 36.55 mm/hr) compared to the infant group (14.05 ± 7.82 10^9^/L, 410.81 ± 178.65 10^9^/L, 53.61 ± 58.92 mm/hr, respectively) (Table 2). Furthermore, incidences of sepsis (*p* = 0.0102), pneumonia (*p* < 0.0001), and septic arthritis (*p* < 0.0001) were significantly higher in neonates compared to infants.

**Table 2:** Comparison between incidences of complications and blood markers of neonates and infants with osteoarticular infections.

***n* (%)**	**Infant (*n*=415)**	**Neonate (*n*=94)**	**Relative Risk**	***P*-value**^a^
**Sepsis**	132 (31.8)	43 (45.7)	1.609 (1.119 – 2.302)	0.0102
**Pneumonia**	32 (7.7)	20 (21.3)	2.375 (1.555 – 3.457)	<0.0001
**Localized cellulitis**	24 (5.8)	4 (0.8)	0.7635 (0.2997 – 1.727)	0.5575
**Septic Arthritis**	194 (46.8)	80 (85.1)	4.901 (2.894 – 8.402)	<0.0001
**Required surgery**	316 (76.1)	74 (78.7)	1.129 (0.7319 – 1.781)	0.5937
**WBC (10^9^/L)**	14.05 ± 7.82	14.91 ± 5.76		0.0153
**PLT (10^9^/L)**	410.81 ± 178.65	475.50 ± 191.42		0.0006
**CRP (mg/L)**	53.61 ± 58.92	32.34 ± 36.55		0.0022
**ESR (mm/hr)**	53.27 ± 114.8	44.35 ± 32.16		0.1766
**Henan Province (*n*=124)**				
	**Infant (*n*=77)**	**Neonate (*n*=47)**	**Relative Risk**	**P-value**
**Sepsis**	25 (31.7)	10 (21.3)	0.6873 (0.3753 – 1.170)	0.1792
**Pneumonia**	12 (15.2)	7 (14.9)	0.9671 (0.4840 – 1.675)	>0.9999^b^
**Localized cellulitis**	9 (11.4)	4 (8.5)	0.7943 (0.3191 – 1.581)	0.7647^b^
**Septic Arthritis**	54 (68.4)	42 (89.4)	2.564 (1.224 – 5.973)	0.0088
**Required surgery**	59 (74.7)	39 (83.0)	1.357 (0.7721 – 2.639)	0.3164
**WBC (10^9^/L)**	15.25 ± 7.37	14.19 ± 5.93		0.8324
**PLT (10^9^/L)**	475.57 ± 188.71	490.02 ± 203.99		0.8304
**CRP (mg/L)**	35.53 ± 44.64	29.37 ± 31.64		0.7119
**ESR (mm/hr)**	49.54 ± 36.78	55.48 ± 36.57		0.3796
**Hebei Province (*n*=346)**				
	**Infant (*n*=302)**	**Neonate (*n*=44)**	**Relative Risk**	**P-value**
**Sepsis**	104 (34.4)	33 (75.0)	4.577 (2.432 – 8.680)	<0.0001
**Pneumonia**	17 (5.6)	13 (29.6)	4.417 (2.529 – 7.232)	<0.0001^b^
**Localized cellulitis**	12 (4.0)	0 (0)	0 (0 – 1.837)	0.3760^b^
**Septic Arthritis**	170 (56.3)	36 (81.8)	3.058 (1.505 – 6.327)	0.0013
**Required surgery**	228 (75.5)	34 (77.3)	1.090 (0.5788 – 2.113)	0.7974
**WBC (10^9^/L)**	14.16 ± 7.92	16.36 ± 4.93		0.0007
**PLT (10^9^/L)**	400.27 ± 172.83	445.20 ± 172.53		0.0244
**CRP (mg/L)**	58.43 ± 62.89	37.12 ± 41.97		0.0769
**ESR (mm/hr)**	54.40 ± 133.80	31.03 ± 18.12		0.0014

a All categorical variables were compared based on the Chi-square test and all numerical variables were compared using the Mann-Whitney U test unless otherwise indicated.

b Fisher's exact test.

These trends were completely negated when the statistics were separated by region. Infants and neonates showed no significant difference in blood markers in Henan province. However, in Hebei province, neonates had significantly higher WBC and PLT, but significantly lower CRP and ESR. Additionally, neonates in Hebei had a significantly higher percentage of pneumonia and sepsis, whereas this was not the case in Henan.

### *Bacterial profile*

**Figure 2:**
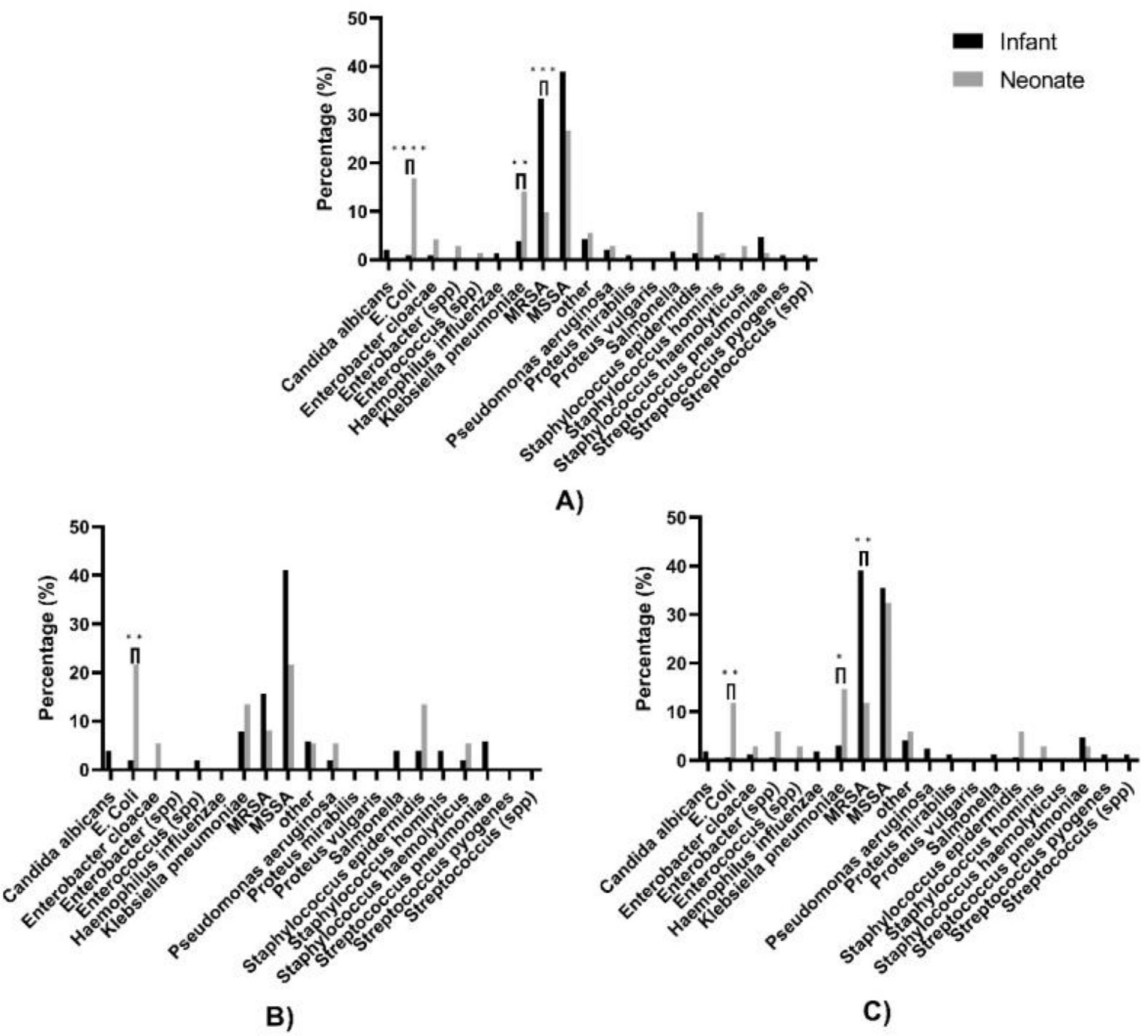
Bacterial profile based on samples from neonates and infants with osteoarticular infections; A) All patients, B) patients from Henan province, C) patients from Hebei province.

Among neonates, 1/94 (1.1%) patients were infected with multiple strains of bacteria and 6/415 (1.4%) infants were found to be infected with multiple strains of bacteria. The three most common bacteria for neonates were methicillin-susceptible *S. aureus* (MSSA) (26.8%), *Escherichia coli* (16.9%), and *Klebsiella pneumoniae* (14.1%), whereas MSSA (38.9%) and methicillin-resistant *S. aureus* (MRSA) (33.3%) were the most common bacteria for infants (Figure 2A). *S. aureus* infections were more prominent in infants (72.2%) compared to neonates (36.6%) (*p* < 0.0001), which was mainly due to the higher number of MRSA infections in infants (33.3%) compared to neonates (9.9%) (*p* < 0.0001). Additionally, a significantly higher percentage of *E. coli* cases (16.9%) and *K. pneumoniae* cases (14.08%) were found in neonates compared to infants (0.9%; *p* < 0.0001 and 3.85%; *p* = 0.0039, respectively).

When examined by province (Figure 2B, C), Hebei province had a higher percentage of MRSA infections in infants (39.1%) whereas Henan province had a relatively lower percentage of MRSA infections in infants (15.7%). Furthermore, the percentage of neonatal *E. coli* cases in Henan (21.6%) was higher compared to Hebei (11.8%). Overall, *S. aureus* was a more common bacteria in both infants (74.6%) and neonates (44.2%) in Hebei as compared to 56.9% in infants and 29.7% in neonates in Henan.

## Discussion

In the present study, some expected trends were found among the neonatal and young infant groups. The frequency of males with osteomyelitis was higher compared to females and incidences of fever were significantly lower in neonates. When examining both Henan and Hebei regions, trends that generally held true in both provinces consisted of neonates having significantly less fever, neonates displaying higher incidences of localized swelling, neonates significantly more likely to develop septic arthritis, neonates having more infections at the humerus, *E. coli* being a higher risk for neonates, and *S. aureus* being more common in infants.

The higher male/female ratio in this study matches that of other epidemiological studies conducted on both young and older children with osteomyelitis.^4^^,^^16^^,^^17^ A commonly proposed theory for the high male/female ratio is that boys tend to be more active, hence being at higher risk of injury.^16^ However, given that the population demographics in the present study were conducted on functionally dependent infants, susceptibility to injury due to increased activity was not a plausible explanation for the higher relative risk in males. One possible explanation consists of genetic differences between males and females, as there are numerous genes that can affect bacterial osteoarticular infections,^18^ although additional investigation is required to determine if genetic variants in any of these genes differ by sex. It should also be noted that males and females also have different innate immune systems that respond differently to the same stimuli.^19^^,^^20^ These innate differences are likely a more plausible explanation for the difference in susceptibility to osteoarticular infections between males and females.

In descending order, the most common regions of infections were the femur, hip, humerus, shoulder, and knee in neonates. Whereas infants were mostly infected in the femur, tibia, hip and humerus. Based on another report,^10^ the most common sites for osteomyelitis in neonates and infants consisted of the femur and humerus, which falls in line with the present study. Another study involving 34 neonates with 42 sites of infection found that infections of the hip (19/42, 45.2%) were the most common.^13^

Based on the overall data, common signs of osteoarticular infections in both neonates and infants included localized swelling (88.7–98.9%), lack of spontaneous movement (79.8–95.7%), and warmth at the site of infection (69.2–85.1%). These manifestations tend to appear as the disease progresses, but swelling and warmth at the site may be difficult for clinicians to detect due to the elevated amount of fat surrounding the limbs of neonates and infants.^21^ Furthermore, detecting apparent pain and the lack of spontaneous movement relies on the observation skills of the physician. In this study, neonates had a significantly lower chance of developing a fever compared to infants, with an incidence rate slightly less than 50%. These results are similar to that of a study on osteoarthritis in neonates where 50% of their patients displayed a fever.^14^ The comparatively lower incidences of fever are thought to be due to neonates having a poorly developed immune system. Fever is generally one of the main symptoms displayed by children with osteoarticular infections and the absence of a fever can often lead to delayed diagnoses and treatments.^10^^,^^16^^,^^22^

In the present study, neonates had significantly higher rates of localized swelling (98.9%) compared to infants (88.7%). Results for the occurrence of localized swelling can likely vary as one study found that 17/17 (100%) neonates with osteomyelitis displayed swelling,^10^ whereas another study only found that 58/77 (75%) of neonates with osteoarthritis displayed signs of swelling.^14^ A possible reason that there was a higher percentage of neonates with signs of swelling in this study could be due to infants increasingly developing body fat,^23^ making it slightly more difficult to observe localized swelling. Higher incidences of septic arthritis were also noticed among neonates in comparison to infants. The frequent co-existence of septic arthritis and osteomyelitis in neonates is thought to be caused by the blood supply from the metaphyseal vessels to epiphyseal vessels in the cartilaginous precursor of the ossific nucleus.^14^^,^^24^

A notable difference in the present results was the higher incidence of sepsis in neonates in Hebei province (75.0%) compared to Henan province (21.3%). The high levels of WBC among neonates in Hebei province also coincide with the high rate of sepsis, to which WBC is often used as a partial indicator for sepsis. Differences in these laboratory blood markers and complications could be due to the difference in bacterial distribution or strains among regions and between neonates and infants. Bacteria strains that are particular to Hebei may induce more severe responses in neonates than infants.

Limitations in this study include the lack of data collected on the outcome, treatment regimen, and follow-up information on these patients. Although this is the largest collection of data on neonatal osteomyelitis and septic arthritis in literature as known to the authors, a larger sample size representing each region of the country would be ideal to provide a clearer epidemiological analysis. Furthermore, this was a retrospective study, and more detailed data, such as bacterial strains and drug resistance were not accounted for. Thus, future studies can be conducted to evaluate the impact of the treatment method used, patient outcomes and prognosis, and bacterial characteristics.

In conclusion, neonates have unique etiologic and pathophysiologic characteristics of osteomyelitis. The signs and symptoms of osteomyelitis in neonates and infants often are subtle and can be easily missed. Several trends and differences were found between neonates and infants with bone or joint infections. Key differences consisted of neonates having significantly lower incidences of fever, a higher incidence of septic arthritis, and higher incidences of localized swelling. Neonates were also infected at the humerus at a significantly higher percentage. Neonates tend to be subjected to a wider distribution of bacterial infections aside from *S. aureus*, including *E. coli* and *K. pneumoniae*. Improved awareness of the clinical characteristics of osteomyelitis and septic arthritis in neonates and infants can allow for timely diagnosis and proper management.

## Authors’ contributions

Conceptualization: Keming Sun, Huiqing Sun, Weyland Cheng; Formal analysis and investigation: all authors; Writing - original draft: Keming Sun, Huiqing Sun, Weyland Cheng; Writing - review and editing: all authors; Funding acquisition: Huiqing Sun, Weyland Cheng.

## Funding

This work was supported by the Department of Science and Technology of Henan Province of China [grant number 162102310001]; and the Joint Construction Project of Henan Medical Science and Technology Research Plan [grant number LHGJ20210667].

## Conflicts of interest

The authors declare no conflicts of interest.
